# Sex and Urbanicity Contribute to Variation in Lymphocyte Distribution across Ugandan Populations

**DOI:** 10.1371/journal.pone.0146196

**Published:** 2016-01-05

**Authors:** Prossy Naluyima, Leigh Anne Eller, Benson J. Ouma, Denis Kyabaggu, Peter Kataaha, David Guwatudde, Hannah Kibuuka, Fred Wabwire-Mangen, Merlin L. Robb, Nelson L. Michael, Mark S. de Souza, Johan K. Sandberg, Michael A. Eller

**Affiliations:** 1 Makerere University Walter Reed Project, Kampala, Uganda; 2 Center for Infectious Medicine, Department of Medicine, Karolinska Institutet, Karolinska University Hospital Huddinge, Stockholm, Sweden; 3 U.S. Military HIV Research Program, Walter Reed Army Institute of Research, Silver Spring, Maryland, United States of America; 4 Henry M. Jackson Foundation for the Advancement of Military Medicine, Bethesda, Maryland, United States of America; 5 Uganda National Blood Transfusion Service, Kampala, Uganda; 6 School of Public Health, Makerere University College of Health Sciences, Kampala, Uganda; 7 SEARCH, Thai Red Cross AIDS Research Center, Bangkok, Thailand; University of Maryland School of Medicine, UNITED STATES

## Abstract

Management of patient care and interpretation of research data require evaluation of laboratory results in the context of reference data from populations with known health status to adequately diagnose disease or make a physiological assessment. Few studies have addressed the diversity of lymphocyte subsets in rural and urban Ugandan populations. Here, 663 healthy blood bank donors from semi-urban centers of Kampala consented to participate in a study to define lymphocyte reference ranges. Whole blood immunophenotyping was performed to determine the frequency and absolute counts of T, B, and NK cells using clinical flow cytometry. Results from blood bank donors were compared to a rural cohort from the district of Kayunga and more urban clinical trial participants from the capital city, Kampala. Relationships between hematological and lymphocyte parameters were also explored. In the semi-urban blood donors, females were significantly different from males in all parameters except the frequency of CD8 T and B cells. Females had higher absolute counts of CD4 T, CD8 T and B cells, whereas males had higher NK cell counts. NK cell frequency and counts were significantly higher in semi-urban blood donors, regardless of sex, compared to more urban study participants. CD8 T cell frequency and counts were significantly higher in the blood donors compared to the rural participants, irrespective of sex. Interestingly, basophil counts were positively associated with overall T cell counts. These findings suggest that both sex and level of cohort urbanicity may influence lymphocyte subset distributions in Ugandans.

## Introduction

Sub-Saharan Africa suffers a disproportionally high burden of infectious and parasitic diseases. According to the 2012 World Health Organization (WHO) statistics report [1], Uganda has a high morbidity (incidence rate per 100,000 population) compared to the rest of Africa, for the three major infectious diseases (HIV/AIDS, malaria and tuberculosis). Clinical trials to assess new or improved preventive and therapeutic treatments are increasingly being conducted in this region [2]. Relevant clinical laboratory lymphocyte reference intervals are important to adequately support clinical management of patients, endpoint evaluation of vaccines and other therapeutic studies, as well as accurate characterization of adverse events observed during the course of a clinical trial. Due to insufficient healthcare infrastructure, Africa is still largely dependent on technology from western countries and limited by transference of clinical laboratory reference ranges not evaluated for the relevant populations.

People living with HIV require regular monitoring of peripheral blood lymphocytes, specifically CD4 T cell frequency and absolute counts, in order to inform treatment decisions [3]. Population-specific reference ranges for lymphocyte subsets in healthy volunteers, needed to highlight lymphocyte abnormalities, remain poorly characterized for Uganda. To date there have been no published reports of percentage and absolute numbers of B cell and natural killer (NK) cell lymphocytes in Ugandan populations. Previous studies involved small sample sizes, were done in distinct rural populations, or did not evaluate all the major lymphocyte subsets [4–6]. Using four-color flow cytometry, we determined clinical laboratory lymphocyte reference ranges in healthy Ugandan blood bank donors because they were more likely to be healthy persons at low risk for infectious diseases, and representative of the Ugandan population. However, more advanced phases of clinical trials may involve thousands of participants from different regions of Uganda to account for pathogen transmission probability [7] and also meet sample size targets. We have previously shown that unrelated hematological reference intervals increase the cost and workload of conducting human vaccine trials [8]. Additionally, studies comparing hematological parameters in African show some variability across different populations [9]. We thus compared the clinical laboratory lymphocyte ranges ascertained from healthy blood bank donors from the semi-urban centers of Kampala, to vaccine study participants from urban centers in Kampala, and cohort study participants from the rural district of Kayunga, to assess how well the currently used ranges represented these three cohorts [10].

This study establishes the reference ranges for the percentages and absolute counts of T, B, and NK cell values in anonymous, healthy, adult Ugandan blood bank donors in the Kampala region. In addition, the blood bank interval values were compared to other Ugandan populations used for clinical studies to identify potential differences and determine factors associated with lymphocyte and hematological variability.

## Materials and Methods

Between 2005 and 2006 Makerere University Walter Reed Project (MUWRP) collected residual blood from blood bank participants to establish laboratory lymphocyte reference ranges in a study RV164: “Determination of Clinical Research Reference Ranges Using Anonymous Healthy Ugandan Blood Bank Donors” [8]. Participants in the blood bank study were anonymous Ugandan blood donors recruited from a 50km radius around the capital city of Kampala (considered semi-urban), and were over 18 years of age, with no significant medical history, met the blood bank definition of healthy [11] and were low risk for sexually transmitted diseases. The study received approval from institutional review boards both in Uganda and the United States. The Walter Reed Army Institute of Research (WRAIR) IRB, and the following local Institutional Review Boards approved by the Uganda National Council for Science and Technology (FWA 00001293) approved the conduct of these studies, as specified, including approval of the written informed consent form as per individual study: HIV/AIDS Research Committee/National AIDS Research Committee IRB (FWA IRB00001515) for Protocols RV156, RV172 and RV173; and the Makerere University Faculty of Medicine IRB (FWA IRB00007568) for RV164. All study participants gave written informed consent. Age, sex, date and time of collection, and regional center were the only patient identifiers provided with the blood samples.

Residual blood from blood collection tubing was harvested into EDTA vacutainer tubes and transported at ambient temperature on the same day to the College of American Pathologists (CAP) accredited MUWRP laboratory. All samples were processed within 8 hours of collection. Whole blood was stained with either CD3 FITC, CD8 PE, CD45 PerCP and CD4 APC four-color monoclonal antibody Multitest^™^ cocktail, or CD3 FITC, CD16+56 PE, CD45 PerCP and CD19 APC Multitest^™^ cocktail in BD Trucount^™^ tubes (Multitest^™^ kits, Becton Dickinson Biosciences, San Jose, California) to enumerate T cell, B cell, and NK cell subsets. Each BD Trucount^™^ tube was incubated for 15 minutes at room temperature in the dark and then 450 μl of 10% FACS Lysing solution (BD Biosciences) was used to lyse the red blood cells for 15 minutes in the dark. Samples were analyzed on a 2-laser FACSCalibur using MultiSet software (all from BD Biosciences, San Jose, California). The procedure was quality controlled using commercial reagents at two CD4 T cell count levels, CD Chex Normal and Low standards (Streck laboratories, Omaha, Nebraska), alongside each batch of samples. In parallel, EDTA anticoagulated blood was used for hematology testing using the Coulter AcT5 diff (Beckman Coulter, Fullerton, California) giving a complete blood count with a five-part differential.

Whole blood collected in serum separation tubes was used for HIV, Hepatitis B (Hep B) and Hepatitis C (Hep C) screening as previously described [8]. Briefly, HIV-1 diagnostics was performed using Genetic Systems rLAV ELISA (BioRad Laboratories, Redmond, Washington). Reactive samples were retested with Vironostika HIV-1 Microelisa Systems (Organon Teknika, Durham, North Carolina), and if reactive, confirmed using the Genetic Systems HIV-1 Western Blot (BioRad Laboratories, Redmond, Washington). Hep B diagnostics was performed using the Genetic Systems HBsAg EIA 3.0 (BioRad Laboratories, Redmond, Washington) and confirmed using the Genetic Systems Confirmatory Assay 3.0 (BioRad Laboratories, Redmond, Washington). Anti-Hep C antibody was detected using the Ortho HCV Version 3.0 ELISA Tests System, and confirmed with Chiron RIBA HCV 3.0 SIA (Chiron Corporation, Emeryville, California). Serum pregnancy testing was performed on all females using Wampole PreVue hCG cassettes (Wampole Laboratories, Inc Dist., Princeton, New Jersey). Individuals with evidence of disease or who were reactive for any of the above tests were not included in this study.

To assess if laboratory results from blood bank participants were comparable to results from communities where participants for clinical studies were recruited, we compared lymphocyte and hematological ranges determined from the blood bank study to results from participants in three clinical studies: (a) RV156: “A Phase I Clinical Trial to Evaluate the Safety and Immunogenicity of a Multiclade HIV-1 DNA Plasmid Vaccine, VRC-HIVDNA009-00-VP, in Uninfected Adult Volunteers in Uganda” [12], (b) RV172: “A Phase I/II Clinical Trial to Evaluate the Safety and Immunogenicity of a Multiclade HIV-1 DNA Plasmid Vaccine, VRC-HIVDNA016-00-VP, Boosted by a Multiclade HIV-1 Recombinant Adenovirus-5 Vector Vaccine, VRC-HIVADV014-00-VP in HIV Uninfected Adult Volunteers in East Africa” [13], and (c) RV173: “Cohort Development for a Possible Phase III HIV Vaccine Trial Among the Population Aged 15–49 years of Kayunga District, Uganda” [14, 15]. Participants from the two vaccine trials were healthy Ugandans aged 18 to 50 years from Kampala (a 20km radius from the center of Kampala, considered urban) who were at low risk for HIV infection and healthy according to a physical exam. Pre-vaccination lymphocyte data from 126 RV156 screening participants and 144 RV172 study enrolees were used. Cohort study participants from Kayunga, a rural Ugandan district, were healthy individuals aged 18 to 49 years. Climate and environmental conditions, with the exception of urbanicity, were quite similar between the two Kampala study groups and the Kayunga cohort, approximately 110 km northeast of Kampala. In all studies participants were negative for HIV, Hep B, Hep C and syphilis infection, had no abnormal clinical chemistry or hematology results, and were not pregnant (for the females). Hematological and lymphocyte analysis was performed in the same CAP accredited MUWRP laboratory, using the same methodology as was done for the blood bank study described above [12–15].

Statistical analysis was performed using Graph Pad Prism version 6.0a for Mac OSX (GraphPad Software, La Jolla, California). To determine a reference range interval that included 95% of the population, the values between the 2.5% and 97.5% limits were calculated after excluding those reactive for the tests listed above. The results were stratified by gender. A non-parametric Mann Whitney U test was used to determine any statistically significant differences between men and women. The non-parametric Kruskal-Wallis test, corrected for multiple comparisons using Dunn’s test, was used to determine differences across study cohorts. The Spearman’s rank correlation was used to assess associations between parameters, with rho ≥ 0.25 considered to be biologically relevant. All p values < 0.05 were considered statistically significant.

## Results

### Sample collection and cohort demographics

Six hundred and sixty three donors participating in regular national blood bank collection services on the outskirts of Kampala (a 50km radius) were recruited and included in the determination of reference ranges. 78% of the semi-urban blood bank donors were male with an age range of 18–56 years. The median age for males and females was 24 and 20 years, respectively (Table 1). The urban vaccine trial study participants were similarly distributed with 72% of the 270 participants being male, 18–49 years of age with a median of 25 and 26 years for males and females, respectively. The rural Kayunga cohort had a more balanced sex distribution with 51% of 246 participants being male, with a median of 24 and 29 years (range: 18–48) for males and females, respectively.

**Table 1:** Population demographics.

Population	Number	Dates Enrolled	Rural/ Urban	Location	Gender Distribution (%)	Age range Min-Max (Median)
**Blood bank donors**	663	2005	Semi-urban	Kampala	Male: 520 (78) Female: 143 (22)	Male: 18–56 (24) Female: 18–49 (20)^a^
**Vaccine trial participants**	271	2005–2006	Urban	Kampala	Male: 195 (72) Female: 76 (28)	Male: 18–49 (25) Female: 18–47 (27)
**Cohort participants**	246	2006	Rural	Kayunga	Male: 125 (51) Female: 121 (49)	Male: 18–48 (24) Female: 18–47 (29)

Blood bank donors were used to establish lymphocyte reference ranges, while vaccine trial and cohort development participants were used for comparison.

^a^Females in this group were significantly younger than those of the other groups, p<0.001.

### Lymphocyte subsets differ by urbanicity and sex

The blood bank study recruited an adequate number of participants for each analyte, as suggested by the Clinical and Laboratory Standards Institute (CLSI) [16], and was therefore used to determine clinical laboratory lymphocyte subset mean, median and reference ranges (2.5–97.5 percentile) for both absolute cell counts and frequency of lymphocytes (Table 2). Females had significantly higher percentages than males for most lymphocyte parameters with the exception of the percentage of CD8 and B-cells that were similar. NK cell percentages on the other hand, were significantly higher in men (median of 14% compared to 11% in females, p<0.001). Similarly, women had significantly higher absolute cell counts than men of CD4 T cells (median of 1010x10^3^ versus 877x10^3^ cells/μl, p<0.001), CD8 T cells (median of 579x10^3^ versus 541x10^3^ cells/μl, p<0.05) and B cells (median of 362x10^3^ versus 315x10^3^ cells/μl, p<0.01), whereas men had higher NK cell absolute counts (median of 291x10^3^ versus 263x10^3^ cells/μl, p<0.05). These results reveal substantial sex associated differences in lymphocyte distribution of healthy Ugandans. It is noteworthy that some participants had CD4 T cell absolute counts below the WHO recommended threshold (<500x10^3^ cells/μl) for initiating antiretroviral therapy in HIV infected persons [17].

**Table 2:** Lymphocyte ranges for blood bank donors in Kampala, Uganda.

	Female (n = 143)			Male (n = 520)			Combined (n = 663)		
Test	Median	Mean (StDev)	2.5–97.5% Range	Median	Mean (StDev)	2.5–97.5% Range	Median	Mean (StDev)	2.5–97.5% Range
CD3, %****	72	72 (5.6)	60.2–82	69	69 (7.5)	52–82	70	69 (7.3)	53–82
CD4, %****	43	43 (6.5)	32–55	40	40 (6.6)	28–54	41	41 (6.7)	28–54
**CD8, %**	24	25 (5.8)	14–36	25	25 (5.9)	15–38	25	25 (5.9)	15–37
NK, %****	11	12 (4.4)	4–21	14	15 (7.2)	5–33	13	14 (6.8)	5–32
**B, %**	14	15 (3.4)	9–23	14.5	15 (4.5)	7–24	14	15 (4.4)	7–24
**CD3, cells/**μl****	1747	1780 (554)	924–3160	1506	1569 (537)	786–2681	1535	1615 (547)	806–2955
**CD4, cells/**μl****	1010	1062 (368)	463–2217	877	913 (335)	429–1558	899	945 (348)	430–1759
**CD8, cells/**μl*	579	616 (246)	255–1276	541.5	578 (282)	243–1100	549	586 (275)	248–1147
**NK, cells/**μl*	263	283 (133)	91–638	291	342 (204)	86–897	284	329 (193)	87–860
**B, cells/**μl**	362	378 (142)	141–747	315.5	344 (155)	130–716	326	351 (153)	133–724
CD4/CD8 ratio**	1.7	1.9 (0.7)	0.9–3.6	1.6	1.7 (1.3)	0.8–3	1.6	1.8 (1.2)	0.9–3.2

Females significantly different from males,

*p<0.05,

**p<0.01,

****p<0.0001.

We next stratified the analysis by sex, and compared lymphocyte parameters across the three distinct cohorts. Urban male vaccine study participants had a significantly higher percentage of cells expressing CD3 (median of 73 versus 69%), and CD4 (median of 44 versus 40%), and CD4/CD8 ratio (median of 1.85 versus 1.61) compared to semi-urban male blood bank participants (Table 3, p<0.001, respectively) but significantly lower percentage of NK cells (Table 3, median of 10 versus 14%, p<0.001) and lower absolute counts of cells expressing CD3 (median of 1366x10^3^ versus 1506x10^3^ cells/μl), CD8 T cells (median of 488x10^3^ versus 541x10^3^ cells/μl), NK cells(median of 198x10^3^ versus 291x10^3^ cells/μl), and B cells ([median of 270x10^3^ versus 315x10^3^ cells/μl], Table 3, p<0.01). Men in the rural Kayunga cohort study also had significantly higher percentages of CD4 T cells (median of 70 versus 69%) and CD4/CD8 ratio ([median of 1.81 versus 1.61], p<0.05, respectively) but a significantly lower percentage of CD8 T cells (median of 23 versus 25%). Furthermore, they had lower absolute counts of CD3-expressing cells (1373x10^3^ versus 1506x10^3^ cells/μl), CD8 T cells (median of 455x10^3^ versus 541x10^3^ cells/μl) and NK cells ([median of 259x10^3^ versus 291x10^3^ cells/μl], p<0.05 respectively) than the semi-urban male blood bank participants. Urban male vaccine study participants had significantly higher T cell percentages than men in the rural Kayunga cohort study, but significantly lower NK cell percentage and absolute count (Table 3; p<0.01).

**Table 3:** Comparison of lymphocyte subset frequency and absolute counts in males.

	Semi-Urban (n = 520)			Urban (n = 195)			Rural (n = 125)			
Test	Median	Mean (StDev)	2.5–97.5% Range	Median	Mean (StDev)	2.5–97.5% Range	Median	Mean (StDev)	2.5–97.5% Range	Significant Differences Between Groups
**CD3, %**	69	69 (7.5)	52–82	73	72 (6.8)	56–83	70	69 (7.7)	48–82	****SU vs U and ***U vs R
**CD4, %**	40	40 (6.6)	28–54	44	44 (6.6)	30–57	41	42 (6.9)	29–57	****SU vs U, *SU vs R, and *U vs R
**CD8, %**	25	25 (5.9)	15–38	25	25 (6.1)	14–39	23	25 (6.3)	12–36	**SU vs R and *U vs R
**NK, %**	14	15 (7.2)	5–33	10	11 (5.5)	5–29	13	14 (6.9)	4–31	****SU vs U and **U vs R
**B, %**	15	15 (4.5)	7–24	14	15 (4.3)	8–25	16	16 (4.1)	8–24	ND
**CD3, cells/**μ**l**	1506	1569 (537)	786–2681	1366	1436 (426)	822–2644	1373	1434 (484)	668–2717	**SU vs U and *SU vs R
**CD4, cells/**μ**l**	877	913 (335)	429–1558	862	871 (260)	465–1469	847	875 (303)	393–1733	ND
**CD8, cells/**μ**l**	542	578 (282)	243–1100	488	502 (193)	228–1036	455	489 (218)	190–971	***SU vs U and ****SU vs R
**NK, cells/**μ**l**	291	342 (204)	86–897	198	232 (152)	69–609	259	291–249	68–583	****SU vs U, *SU vs R, and **U vs R
**B, cells/**μ**l**	316	344 (155)	130–716	270	302 (143)	117–718	311	326 (137)	125–720	***SU vs U
**CD4/CD8 ratio**	1.6	1.7 (1.3)	0.8–3.0	1.9	1.9 (0.6)	0.8–3.3	1.8	2.0 (0.7)	0.9–3.9	***SU vs U and ***SU vs R

Males002C SU = Semi-Urban, U = Urban, R = Rural,

*p<0.05,

**p<0.01,

***p<0.001,

****p<0.0001,

ND = no difference.

The non-parametric Kruskal-Wallis test, corrected for multiple comparisons using Dunn’s test, was used to determine differences across groups.

Interestingly, women displayed fewer differences across the three cohorts compared to men (Table 4). The urban female vaccine study participants had significantly higher percentage of CD4 T cells (median of 74 versus 72%), but significantly lower percentage and absolute count of NK cells (median of 9 versus 11% and 184x10^3^ versus 263x10^3^ cells/μl, respectively) compared to semi-urban blood bank study female participants (Table 4, all p<0.05). The rural Kayunga cohort female participants had a significantly higher percentage of CD4 T cells (median of 46 versus 43%), higher CD4/CD8 ratio ([median of 2.03 versus 1.71], Table 4, both p<0.05), but lower percentage of CD3-expressing cells (median of 71 versus 72%) and CD8 T cells (median of 22 versus 24%) and CD8 absolute counts ([median of 507x10^3^ versus 579x10^3^ cells/μl], Table 4, all p<0.05) compared to semi-urban blood bank study female participants. Additionally, women from the rural Kayunga cohort study had a significantly lower percentage of CD8 T cells (median of 22 versus 24%), but significantly higher NK cell percentage (median of 11 versus 9%) and absolute counts ([median of 258x10^3^ versus 184x10^3^ cells/μl], Table 4, all p<0.05) compared to urban female vaccine study participants. Taken together, these data reveal significant differences in female lymphocyte subset distribution across cohorts with varying levels of urbanicity.

**Table 4:** Comparison of lymphocyte subset frequency and absolute counts in females.

	Semi-Urban (n = 143)			Urban (n = 76)			Rural (n = 121)			
Test	Median	Mean (StDev)	2.5–97.5% Range	Median	Mean (StDev)	2.5–97.5% Range	Median	Mean (StDev)	2.5–97.5% Range	Significant Differences Between Groups
**CD3, %**	72	72 (5.6)	60.2–82	74	73 (5.5)	58–81	71	71 (7.6)	53–83	*U vs R
**CD4, %**	43	43 (6.5)	32–55	45	45 (5.7)	35–56	46	45 (7.6)	31–60	*SU vs U and *SU vs R
**CD8, %**	24	25 (5.8)	14–36	24	25 (6.0)	13–39	22	23 (6.4)	13–35	**SU vs R and **U vs R
**NK, %**	11	12 (4.4)	4–21	9	9.8 (4.0)	4–20	11	12 (6.2)	4–26	*SU vs U and **U vs R
**B, %**	14	15 (3.4)	9–23	16	15 (3.7)	9–23	15	16 (4.3)	8–25	ND
**CD3, cells/**μ**l**	1747	1780 (554)	924–3160	1641	1682 (524)	702–3082	1590	1700 (586)	787–3549	ND
**CD4, cells/**μ**l**	1010	1062 (368)	463–2217	1011	1045 (358)	469–2128	1024	1071 (327)	486–1915	ND
**CD8, cells/**μ**l**	579	616 (246)	255–1276	545	574 (223)	194–1107	507	557 (349)	235–1095	**SU vs R
**NK, cells/**μ**l**	263	283 (133)	91–638	185	224 (124)	79–636	258	299 (184)	82–758	***SU vs U and **U vs R
**B, cells/**μ**l**	362	378 (142)	141–747	341	369(154)	127–881	360	379 (176)	137–815	ND
**CD4/CD8 ratio**	1.7	1.9 (0.7)	0.9–3.6	1.9	2.0 (0.7)	1.0–3.9	2.0	2.2 (0.8)	1.1–4.2	***SU vs R

Females, SU = Semi-urban, U = Urban, R = Rural,

*p<0.05,

**p<0.01,

***p<0.001,

ND = no difference.

The non-parametric Kruskal-Wallis test, corrected for multiple comparisons using Dunn’s test, was used to determine differences across groups.

### Hematological parameters differ across populations

**Fig 1:**
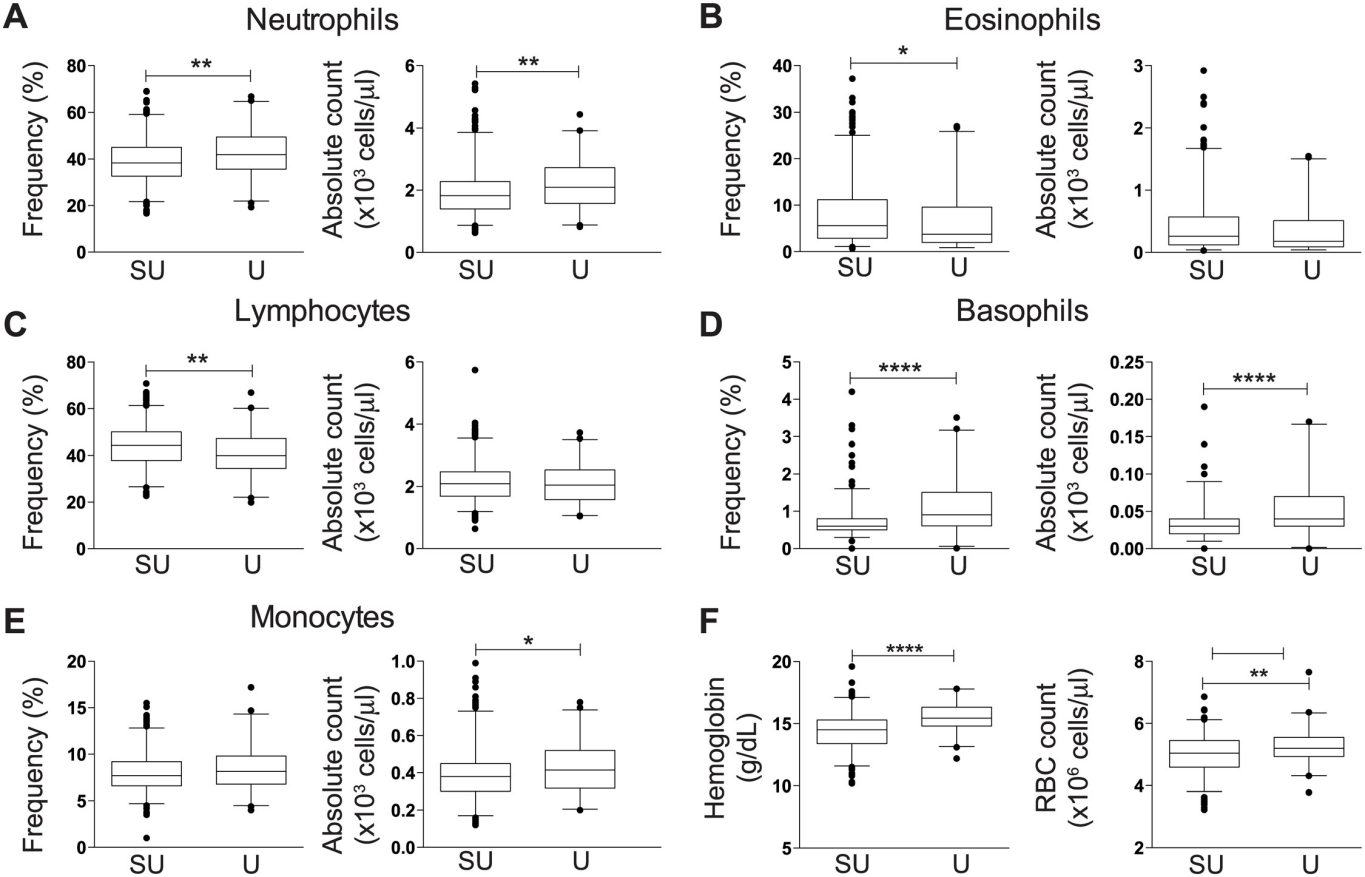
Comparison of hematological parameter frequency and absolute counts in males. Box and whisker plots showing median and 2.5–97.5 percentiles of (A) neutrophils in semi-urban blood bank donors [SU] and vaccine trial [U]; (B) eosinophils; (C) lymphocytes; (D) basophils; (E) B monocytes; and (F) hemoglobin levels and RBC counts. The non-parametric Mann Whitney U test was used to determine any statistically significant differences between cohorts; *p<0.05, **p<0.01, ***p<0.001, ****p<0.0001.

**Fig 2:**
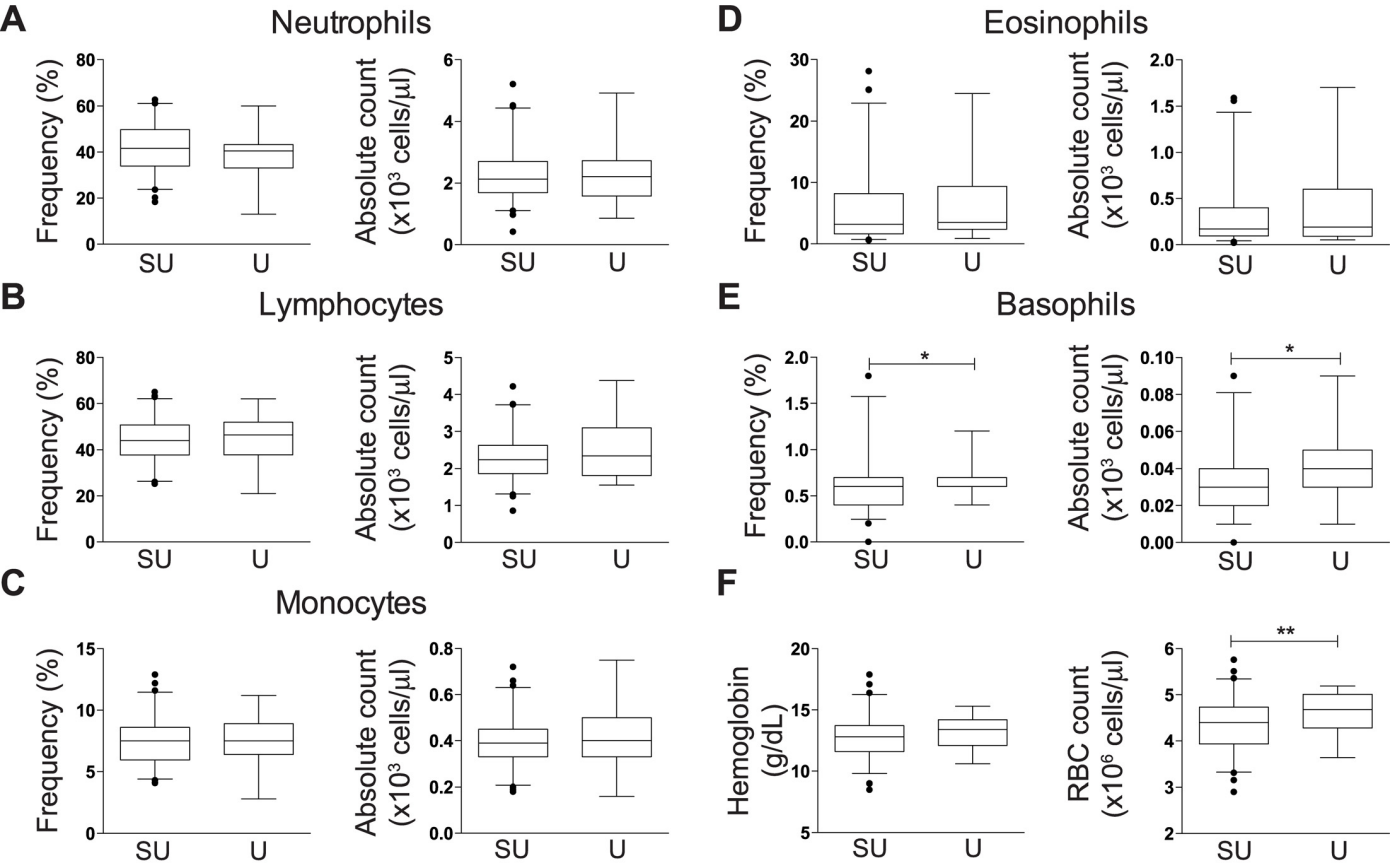
Comparison of hematological parameter frequency and absolute counts in females. Box and whisker plots showing median and 2.5–97.5 percentiles of (A) neutrophils in semi-urban blood bank donors [SU] and vaccine trial [U]; (B) eosinophils; (C) lymphocytes; (D) basophils; (E) B monocytes; and (F) hemoglobin levels and RBC counts. The non-parametric Mann Whitney U test was used to determine any statistically significant differences between cohorts; *p<0.05, **p<0.01, ***p<0.001, ****p<0.0001.

To further investigate differences in participants across geographically disparate sites, we compared hematological parameters between a subset of 637 blood donors and 121 vaccine study participants, for whom this data was readily available. Stratified by gender, 79% and 71% of the semi-urban blood bank donors and urban vaccine study participants were male, respectively. Men participating in vaccine trials had significantly higher percentages of neutrophils (median of 41.9 versus 38.3%) and basophils (median of 0.9 versus 0.6%), and higher absolute counts of all parameters with the exception of lymphocytes (median of 2.05x10^3^ versus 2.09x10^3^ cells/μl) and eosinophils (median of 0.18x10^3^ versus 0.26x10^3^ cells/μl) compared to semi-urban male blood donors (Fig 1; all p<0.05). Percentages of lymphocytes and eosinophils were significantly lower in urban men participating in the vaccine trials compared to male blood donors (median of 39.9 versus 44.4% and 3.75 versus 5.6%, all p<0.05). Consistent with lymphocyte subset parameters, women had fewer hematological differences between cohort populations compared to male counterparts, with women participating in vaccine trials having higher basophil percentage and absolute counts (median of 40 and 30 cells/μl, respectively) and red blood cells ([RBC], median of 4.4x10^6^ and 4.68x10^6^ cells/μl, respectively) compared to semi-urban female blood donors (Fig 2, all p<0.05).

### Age associates positively with CD4%, red blood cell count, and hemoglobin levels in women

**Fig 3:**
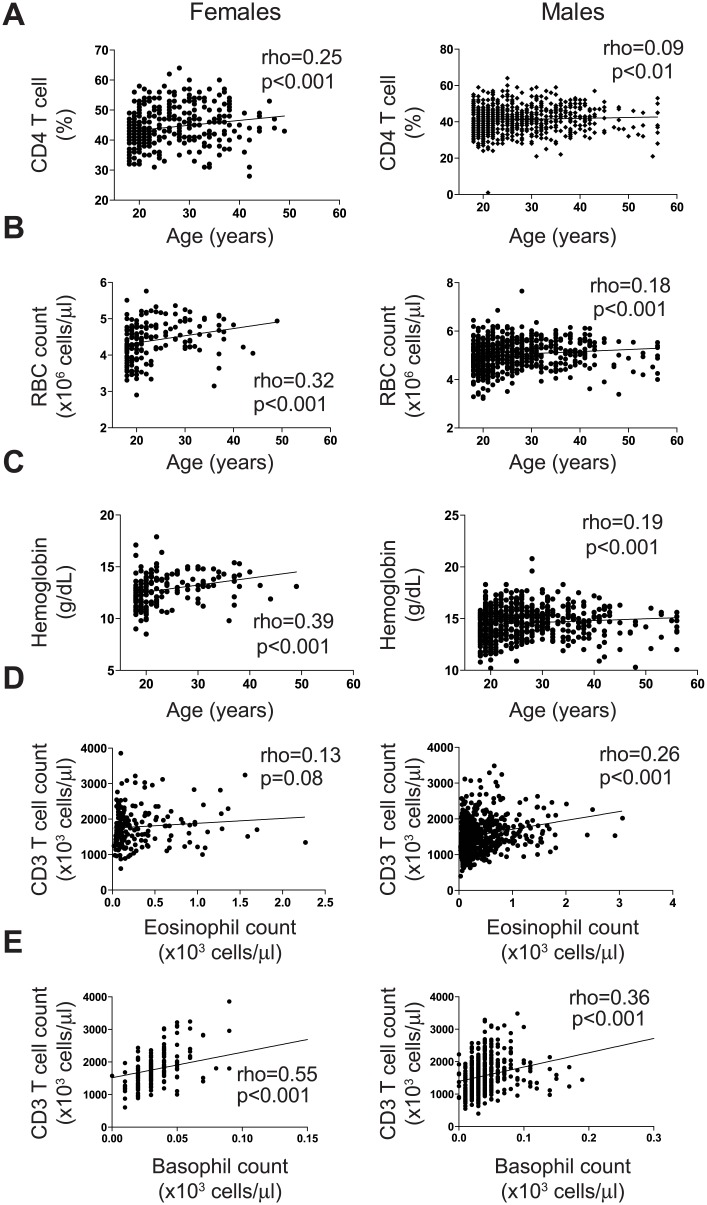
Association between age, lymphocytes and hematological parameters. Correlation graphs of (A) Age and CD4 T cell frequency; (B) Age and RBC count; (C) Age and hemoglobin levels; (D) eosinophil and CD3 T cell counts; and (E) basophil and CD3 T cell counts. The Spearman’s rank correlation was used to assess associations between parameters, with rho ≥ 0.25 and p values < 0.05 considered statistically significant.

We next investigated whether the age of a participant was associated with the lymphocyte and hematological parameters. For this purpose we pooled the data from all cohort studies, and analysed by gender (Fig 3). Age of female participants correlated weakly with the frequency of CD4 T cell lymphocytes (Fig 3A; rho = 0.25, p<0.001), RBC count (Fig 3B; rho = 0.32, p<0.001) and hemoglobin (Hgb) level (Fig 3C; rho = 0.39, p<0.001). No such associations were observed among the men, and none of the other parameters correlated with age in either sex. Despite the few associations observed, our data suggests that age is not the primary factor driving diversity in the lymphocyte and hematological parameters measured within these cohorts.

### Eosinophil and basophil counts associate with CD3 T cell counts

**Fig 4:**
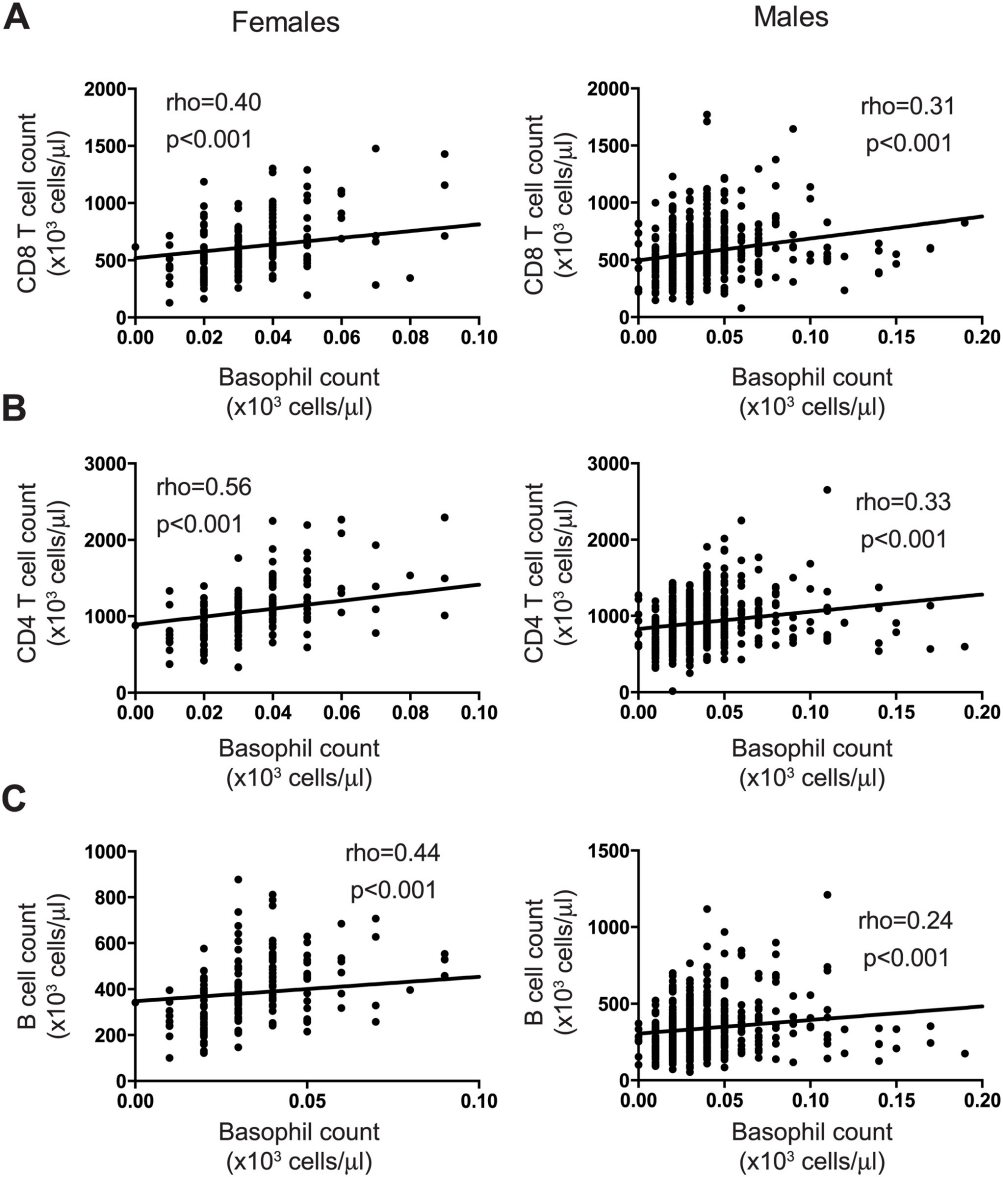
Associations between lymphocyte and basophil counts. Correlation graphs of (A) basophil and CD8 T cell counts; (B) basophil and CD4 T cell counts; and (C) basophil and B cell counts. The Spearman’s rank correlation was used to assess associations between parameters, with rho ≥ 0.25 and p values < 0.05 considered statistically significant.

We further investigated if the hematological data was associated with the lymphocyte data. Again, we combined the participant data from all studies and analysed by sex. In men, eosinophil and basophil counts correlated positively, albeit weakly, with absolute CD3 T cell counts (Fig 3D, rho = 0.26 and 3E, rho = 0.36; p<0.001, respectively), whereas in women, only basophil count correlated positively with absolute CD3 T cell counts (Fig 3E, rho = 0.55, p<0.001). Interestingly, basophil counts were positively associated with the absolute CD4 and CD8 T cell counts regardless of sex, but not with age (Fig 4A and 4B, p<0.001 respectively). In addition, the basophil count correlated positively with the absolute B cell count in women, but not men (Fig 4C, p<0.001). Taken together, our data reveals positive associations between granulocyte and T cell parameters in healthy Ugandans regardless of sex.

## Discussion

Sub-Saharan Africa continues to struggle with a disproportionately high burden of both infectious and parasitic diseases [1]. As a result, clinical trials to assess preventive and therapeutic interventions are increasingly being conducted [2]. It is imperative that data generated are representative of the studied population and interpreted accordingly [16]. Furthermore, understanding differences in the cellular composition of the immune system and thus also immunological reference ranges between groups, regions and countries can help researchers correctly interpret data obtained from studies of infectious diseases, as well as studies of novel vaccines and therapeutic interventions. To this end, we set out to establish clinical laboratory reference ranges for peripheral blood lymphocytes in Ugandans using anonymous healthy blood bank donors. Previous studies were either decades old [5], used dual platform technology thus increasing the margin of error [4], or combined data from different African countries [6]. We show that some healthy participants have ‘low’ CD4 T cell absolute counts, which has public health implications for HIV management [17]. To our knowledge, the present study is the first to report on NK cell and B cell lymphocyte counts in Ugandans.

Interestingly, we found that women had significantly higher lymphocyte frequencies and absolute counts in almost all subsets with the exception of NK cells, where they had less than men. This is in agreement with other studies done in Uganda and elsewhere, for T cell subsets [4–6, 9, 18–21]. Also, immunological differences between male and female children have previously been observed, albeit not consistently [20–23]. The underlying basis for this sex difference has not been elucidated, however variance in the normal ranges of lymphocyte subsets may contribute to differential immune responses to infection, vaccination, or treatment. For example, men and women display differences in immune response to vaccination [24], susceptibility to infectious and parasitic diseases [25, 26], immune responses to infection [27, 28] and treatment [29], immune-mediated pathology and autoimmune disease [25, 30, 31] and mortality [32]. These differences involve both innate and adaptive immune cells, and vary according to the geographic location of the study, suggesting that the sex differences observed in lymphocyte subsets may have clinical significance.

Currently, several HIV therapeutic clinical trials are being conducted in more than one region of Uganda [2]. The successful evaluation of clinical interventions requires clinical laboratory ranges that reflect the population in which these interventions are targeted. We compared lymphocyte data obtained from healthy blood bank donors to data generated from two other studies conducted in different Ugandan locations. Surprisingly, not only were there significant lymphocyte subset differences between the rural (Kayunga) and urban (Kampala) populations, but the blood bank donors, a semi-urban population, demonstrated significantly different lymphocyte frequencies compared to urban vaccine study participants. Additionally, urban vaccine study participants demonstrated higher granulocyte counts. Here we show that, consistent with other studies in Africa [6, 21, 23, 33], and elsewhere [21, 34] populations in different geographic locations may have statistically significant differences in lymphocyte and other hematological parameters. The reason for this variation across Ugandan populations remains unclear. Possible factors include diurnal variation, altitude, nutrition, socio-economic status, environment [6, 18, 20, 33, 35], genetics [22, 36] or seasonal effects [37]. Kayunga is located 110 km northeast of Kampala but is only 120 m lower in altitude (1070 m versus 1190 m, respectively) and both are malaria-endemic areas, thus these factors are unlikely to influence these results. Peripheral blood T cell counts have been shown to be stable during the day [38] while seasons have minimal to no impact [37]. Notably, all three populations were heterogeneous as regards to tribe, and thus genetics. Additionally, vaccine study participants achieved consistently higher education levels compared to blood bank donors living in semi-urban Kampala areas, where both lower and higher educated individuals were observed. The rural cohort in Kayunga mostly enrolled participants with limited education and menial jobs. Therefore nutrition, socio-economic status and composition of the intestinal microflora may contribute to the differences in peripheral blood indices, despite geographical similarity between these groups [4, 18, 22]. Future studies should explore the relative impact of environmental factors to better understand the mechanistic basis in immune cell subset variation. Additionally, incorporating a more detailed health evaluation and history of individuals, in the context of the immunologic differences, should be considered to assess the clinical relevance of our observations.

It was interesting to find that in women, the frequency of CD4 T cells, RBC count and Hgb levels increased modestly with age, whereas in males there was no such relationship. This is in contrast with other studies in Uganda [4] and Western Kenya [19], particularly in males, which underscores the differences in populations at different locations. It has been shown that sex hormones play a role in immune responses and influence the outcome of infection [27]. Blood loss due to menstruation [39], sub-optimal nutrition and pregnancy [40] could possibly contribute to these age patterns in women. Das *et al* recently found that iron deficiency was associated with lower CD4 T cell counts in children in India [41]. Notably, we did not find an association of CD4 T cell counts with either RBC counts or Hgb levels. Further work is needed to elucidate the observed CD4 T cell and age association.

Granulocytes are studied for their role in allergic conditions, in addition to immune defence against parasitic infections [42] common to sub Saharan Africa [6]. To our knowledge, the present study is the first to show a positive correlation between eosinophil and basophil counts with T and/or B cell counts. It is becoming more apparent that granulocytes, through their innate production of cytokines, play important roles not only in host defence but also in directing and regulating adaptive immune responses [42–44]. A detailed study to elucidate these relationships in the context of populations with high parasitic burden is warranted.

Our study, using heterogeneous populations of blood bank donors, vaccine study volunteers, and research cohort participants, has evaluated factors that contribute to lymphocyte distribution in Ugandans, to include T cells, NK cells and B cells. We describe significant differences between the sexes in most lymphocyte subsets with women having more CD4 T cells and men having higher levels of NK cells. Furthermore, we have shown that even within the same country there are differences in lymphocyte and hematological parameters between cohorts in different locations. A limitation of our study is that we did not screen for all the medical conditions that could impact on the laboratory data, although we followed the procedure used to screen potential volunteers for clinical trials in Uganda [10]. Increasing epidemiological evidence supports the notion that sex and geographic location may impact morbidity and mortality in disease and immune response to vaccination [32, 35, 36]. Well-controlled biological studies are needed to definitively answer this question, so as to guide both clinical trial design and public health strategies.
